# Case Report: Primary cardiac synovial sarcoma invading the tricuspid valve in a pregnant woman

**DOI:** 10.3389/fcvm.2024.1437903

**Published:** 2024-10-18

**Authors:** Mixia Li, Maoxun Huang, Hulin Piao, Yong Wang, Kexiang Liu

**Affiliations:** Department of Cardiovascular Surgery, The Second Hospital of Jilin University, Changchun, China

**Keywords:** synovial sarcoma, cardiac, pregnant woman, surgery, cardiopulmonary bypass, fetal outcomes

## Abstract

Primary cardiac synovial sarcoma (PCSS) is a rare and highly aggressive tumor with a significant mortality rate. Treatment guidelines have not been defined given the relative rarity of the condition, especially for pregnant women. Described herein is a 36-year-old pregnant woman at 29 weeks with gestation who was hospitalized due to chest tightness and nausea, and echocardiography found a mass involved in the right heart and the tricuspid valve. She had to undergo cardiac surgery because the mass almost blocked the opening of the tricuspid valve. She underwent a radical resection of the masses and tricuspid valve, followed by replacement of the tricuspid valve with a mechanical valve. She successfully delivered a healthy baby boy. The diagnosis of synovial sarcoma is confirmed by positive results indicating rearrangement of the SYT gene. The patient survived throughout the 30-month follow-up period. There are no reported cases of pregnant women diagnosed with cardiac synovial sarcoma and have undergone cardiac surgery and cesarean section. Our treatment plan not only maximizes patient survival but also ensures fetal survival. This situation is rare and needs documentation.

## Introduction

Synovial sarcoma is an aggressive malignant soft tissue tumor derived from primitive pluripotent mesenchyme capable of epithelial differentiation presenting in adolescents and young adults, with approximately 80% originating adjacent to the joints or tendon sheaths of the extremities (1). Primary cardiac synovial sarcomas (PCSS) represents an exceptionally rare subset. We reviewed the literature and found 52 cases of PCSS originated from the cardiac chambers or myocardium. Notably, There are no documented cases of PCSS in pregnant women. Surgical resection remains the first-line treatment, however, recurrence rates remain high due to incomplete macroscopic resection achieved in approximately one-third of patients (2). The need for cardiac surgery during pregnancy is rare, only 1% to 4% of pregnancies are complicated by maternal cardiac disease (3). Performing cardiac surgery on cardiopulmonary bypass (CPB) during pregnancy is high-risk for both the fetus and the mother.

## Case presentation

**Figure 1:**
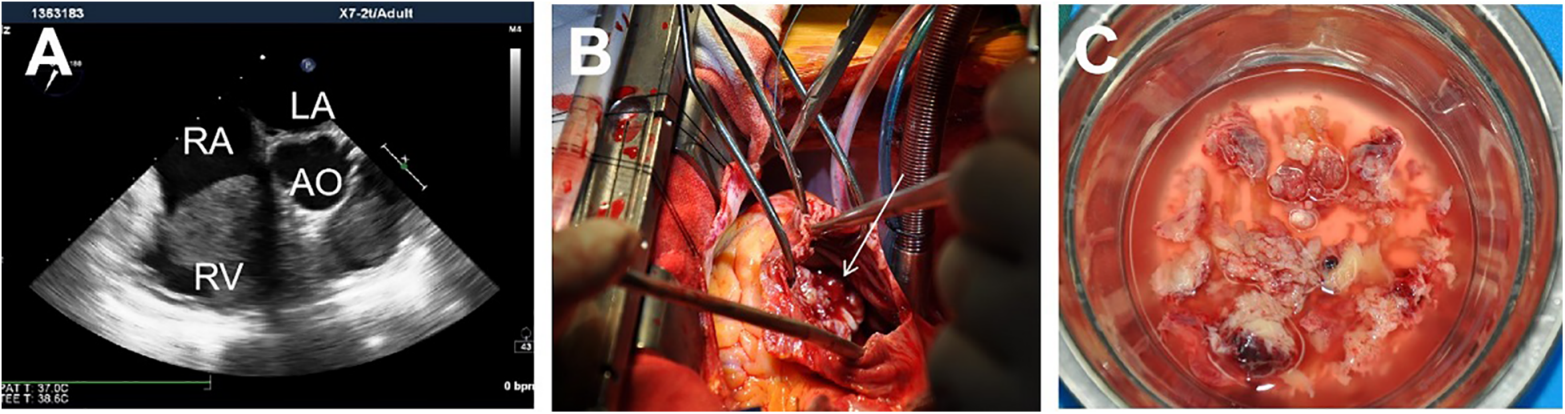
A mass adherent to the right heart (**A**); Intraoperative view showing the mass almost occupies the opening of the tricuspid valve (**B**). The characteristics of the gross specimen are soft, gray white, friable (4) (**C**).

A 36-year-old pregnant woman at 29 weeks gestation was admitted with complaint of chest tightness and nausea persisting for 3 days. Echocardiography confirmed the presence of an irregular mass measuring 85 mm × 40 mm arising from the tricuspid annulus, extending from the inlet to the outflow tract of the right ventricle (Figure 1A). Abdominal color ultrasound showed the congestion liver. Obstetric evaluation showed a uterine height of 30 cm and abdominal circumference of 90 cm; Biparietal diameter of the fetus (BPD) is 74 mm; Head circumferece (HC) is 277 mm; Abdominal circumferece (AC) is 248 mm; Femur length (FL) is 54 mm; The fetal heart rate was recorded as 152 beats/min. Because of her pregnancy, she did not undergo any other radiation tests.

After consultation with cardiologists, obstetricians, anesthesiologists, and perfusionists, we have made the decision to proceed with surgical intervention. The initial option entails tumor removal on CPB without inducing cardiac arrest while maintaining the pregnancy. Alternatively, the second option involves tumor excision on CPB under cardiac arrest and termination of the pregnancy.

After general anesthesia, median sternotomy was performed. After full heparinization, CPB was instituted through cannulations of the aorta, inferior vena cava, and superior vena cava. On atriotomy, the mass was almost occupying the opening of the tricuspid valve (Figure 1B). It was attached to the root of the anterior leaflet and septal leaflet, destroying the tricuspid valve entirely. We decided to remove the tumor and replace the tricuspid valve under cardiac arrest. Cold cardioplegic solution was instilled via the aortic root after cross clamping the aortic. Simultaneously, it was crucial to draw cardioplegia from the coronary sinus orifice. Remove the mass and irrigate the cardiac chambers thoroughly (Figure 1C). The valve has replaced with a 29-mm mechanical valve. The aortic clamp was opened and right atrium was sutured. After careful hemostasis and closing the wound in layers, The rest of surgery was completed routinely. Subsequently, the patient underwent a cesarean section successfully delivering a healthy baby boy. The CPB time was 74 min and cross clamp time was 36 min.

**Figure 2:**
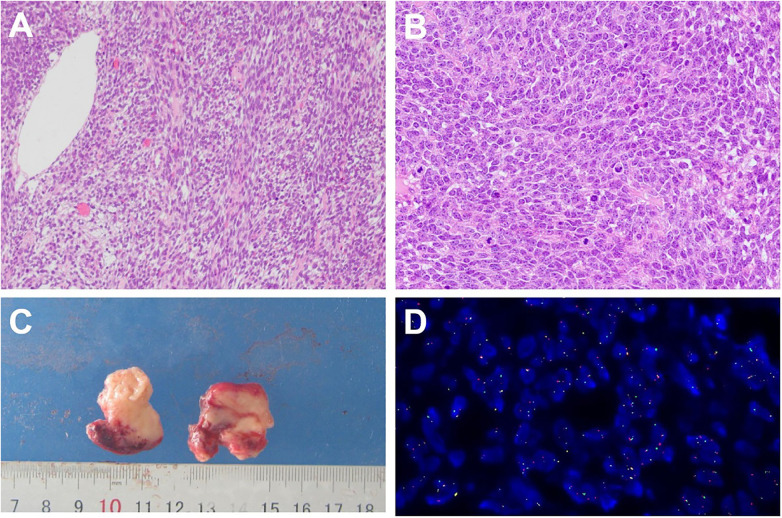
Histology appearance: uniform spindle cells arranged in fascicles. The spindle cells showing eosinophilic cytoplasm and round to oval nuclei.nuclear pleomorphism and with mitoses readily observed (hematoxylin and eosin stain × 100) (**A**); Poorly differentiated region((hematoxylin and eosin stain × 100) (**B**); Detection probe: LBP SYT gene fracture probe; 1G1R1F accounted for 48%; Chromosome site: 18q11. Probe labeling: green signal (**G**) is GSP SYT (Centromere), red signal (**R**) is GSP SYT (Telomere) (**D**).

Subsequently, the postoperative pathology reveals the presence of monophasic synovial sarcoma, specifically confirmed by positive expression of Vimentin, TLE1, and BCL-2 in immunohistochemical analysis. The diagnosis of synovial sarcoma is further supported by positive results indicating SYT gene fracture rearrangement (Figure 2).

She recovered without complication and was discharged on the 7th postoperative day. She received adjuvant chemotherapy consisting of a combination of anlotinib, toripalimab, and eribulin. The patient survived throughout the 30-month follow-up period. However, a echocardiography performed 26 months post-operatively revealed recurrence in the right ventricle and the right ventricular outflow tract. The patient refused to underwent re-operation to remove the mass.

## Discussion

Primary cardiac synovial sarcomas (PCSS) is extremely rare, accounts for approximately 4.2% of all malignant primary cardiac tumors. They most commonly arise from the right atrium (24%) (5). PCSS has a 3:1 male predominance and a mean age of diagnosis in the fourth decade of life (1). These tumors are characterized by a t(X;18)(p11.2;q11.2) chromosomal translocation and the formation of a SS18-SSX fusion oncogene. There are three main histological types of PCSS, monophasic (the most common), biphasic and poorly differentiated (Table 1). In addition to histological characteristics, molecular demonstration of SYT gene rearrangement is essential in confirming the diagnosis (4). Complete resection is the treatment of choice, with additional chemotherapy and/or radiation therapy, when necessary. However, Cardiac surgery is inherently dangerous for both, the mother and fetus with mortality rates near 10% and 30%, respectively (3).

**Table 1:** Pathologic features.

	Pathologic features of PCSS
Gross	The gross pathology reveals pink-tan, fleshy mass. The cut section is usually uniform, pale brown or gray, and often is relatively soft and friable because of the paucity of intercellular strorna.
Microscopy	There are three main histological types of synovial sarcomas (SS), monophasic (the most common), biphasic and poorly differentiated. Monophasic SS is composed of highly cellular solid sheets or fascicles of remarkably uniform small spindle cells with a high nuclear-to-cytoplasmic ratio. Biphasic SS contains distinct but intermingled epithelial and spindle cell components. Poorly differentiated synovial sarcoma is composed of solid sheets of uniform, closely packed, relatively small round-to-short spindled cells, often with a prominent hemangiopericytoma-like vasculature. Poorly differentiated areas may be found focally in up to 20% of otherwise typical monophasic or biphasic SS.
Immunohistochemistry	The lesional cells of monophasic SS are positive for EMA, cytokeratins and TLE1. Up to two thirds of synovial sarcomas express CD99, Bcl-2 positivity is seen in most cases. The glandular component of biphasic SS is uniformly and strongly positive for keratins and EMA.
Molecular Genetics	The gold standard for diagnosis of SS is a t (X;18) balanced translocation, which involves the SS18 (SYT) gene on chromosome 18 and either the SSX1 or the SSX2 gene on chromosome X.

Given the rarity and heterogeneity of PCSS cases, there are no established treatment guidelines. Complete surgical resection is the best treatment choice for cardiac sarcomas. With the advantages of less trauma, less bleeding, and short hospital stay, minimally invasive is gaining popularity in the treatment of cardiac tumors. Moscarelli et al. (6) found that there was no difference in the occurrence of postoperative adverse cardiac events and neurological events between median sternotomy and minimally invasive. Considering that our patient is pregnant and the protection of the fetus, we needed to shorten the operation and CPB time as much as possible. Finally, we performed median sternotomy. Moreover, adjunctive radiotherapy and chemotherapy are associated with greater survival, and the most commonly used chemotherapy regimen is ifosfamide and doxorubicin (1, 2). Our patient received adjuvant chemotherapy consisting of a combination of anlotinib, toripalimab, and eribulin.

After an extensive review of the literature, no documented cases exist of PCSS occurring in pregnant women and being treated with surgery (Table 2). Cardiac surgery carried out on CPB in a pregnant woman is associated with poor neonatal outcomes although maternal outcomes are similar to cardiac surgery in non-pregnant women. Fetal mortality rates associated with maternal cardiac surgery during pregnancy range from 20% to 30% (3). Sustained forceful uterine contractions during CPB are considered as the most important contributors to fetal death (7). To mitigate this risk, we prioritize maintaining high perfusion flow rates and normothermia. Additionally, cardioplegia administration may elevate serum potassium levels, potentially leading to fetal hyperkalemia and subsequent cardiac arrest. To prevent this complication, we carefully avoid any entry of cardioplegia into the bloodstream by discarding it through the coronary sinus orifice. Our patient was at 29 weeks gestation. Considering that fetal development is acceptable, we performed a cesarean section. Primary cardiac sarcomas of all types have poor outcomes, PCSS is no different, with a mean survival of 9 to 16.5 months (8). As of now, our patients have survived for 30 months after surgery and the baby boy is also alive and well.

**Table 2:** The prior case reports of cardiac synovial sarcoma.

No.	Year	Author	Age, Y/Sex	Location	Size (cm)	Histology	Treatment	Clinical outcome
1	1978	McAllister HA	30/M	RV/pericardium	_	_	Autopsy finding	
2	1988	Sheffield EA	53/M	RA/LA	_	_	Surgery	6 mo, DOD
3	1990	Siebenmann	31/F	RA/LA	_	_	Heart transplant	3 mo, DOD
4	1992	Burke AP	46/F	LA	_	_	_	_
5			M	_				
6	1994	Karn CM	35/M	RA/pericardium	_	_	Surgery + CT	9 mo, DOD
7	1995	Iyengar V	38/M	RV	_	_	Surgery	12 mo, DOD
8	1997	Nicholson AG	13/M	RA	5	B	Surgery,CT	10 mo, NED
9	1998	Fujioka M	29/M	LA	5	_	Surgery	8 mo, DOD
10	1999	Donsbeck AV	34/M	RA	_	_	_	Died
11	1999	Casselman	24/F	LA/MV	5.1	M	Surgery + MVR	4 mo, AED
12	2000	Bittira B	47/M	RA	4.5	B	Surgery + CT	_
13	2003	McGilbray TT	30/M	LV/MV	_	B	Surgery + T	_
14	2004	Hannachi Sassi	45/M	RA	_	_	_	_
15	2004	Hazelbag HM	42/M	LA/MV	_	_	_	1 mo, DOD
16	2005	Miller DV	66/M	MV	5.1	M	Surgery + CT	6 mo, NED
17	2006	Policarpio-Nicolas ML	36/M	LV	4.4	B	_	_
18	2007	Zhao Q	36/M	RA/LA/Interatrial septum	_	m	Surgery	_
19	2007	Boulmay B	19/F	RV	_	_	Surgery	4 mo, AED
20	2008	Kim CH	35/M	RV	4.5			9 mo, DOD
21			53/M	RA	12			10 mo, DOD
22			29/F	RA	7.5			34 mo, NED
23	2009	White RW	22/M	RA	20	_	Surgery + CT	_
24	2010	Lv X	53/F	LV	5.3	_	Surgery	1 mo, DOD
25	2010	Fresneau B	16	RV	_	_	CT	6 mo, DOD
26	2010	Talukder M	_	_	_	_	_	_
27	2011	Yokouchi Y	51/M	LA/Pericardial	6	M	Surgery + RT	9 mo, NED
28	2014	Wolf M	61/M	RV	10	_	CT	_
29	2014	Khan I	_	RV	_	_	_	_
30	2015	Huo Z	51/F	RA-RV/TV	8	B	Surgery + CT	_
31	2015	Stine JG	60/M	RA	10.6	_	_	Dead
32	2015	Sharma A	26/F	LA	7	_	Surgery	1 mo, AWD
33	2015	Prifti E	11/M	MV	1.1	_	Surgery + MVR	12 mo, NED
34	2015	Eswaran P	35/M	RA	6.3	B	Surgery + CT	24 mo, NED
35	2017	Hosseinzadeh Maleki M	21/F	RA	7.3	_	Surgery + TVR,CT	15 mo, NED
36	2018	Maeda S	19/M	LV	15	M	Surgery + CT + Resugery	36 mo, DOD
37	2018	De Hous N	32/M	RA/Interatrial septum	5.5	B	Surgery	15 mo, NED
38	2018	Braham W	61/F	RV	_	_	Surgery + CT	6 mo, AWD
39	2018	Coli A	52/M	RV/RA	7.1	B	CT + Heart transplant	18 mo, DOD
40	2019	Zhang G	52/M	LA	5	B	Surgery	6 mo, DOD
41	2019	Zhang HK	32/M	RV	7.3	B	Surgery + TVR	12 mo, DOD
42	2020	Akhmerov A	19/M	LV	6.6	_	Surgery + CT	15 mo, NED
43	2021	Shah D	37/M	RA	_	B	Surgery	_
44	2021	Teng F	32/M	RV	7.4	B	Surgery + TVR	12 mo, DOD
45			26/M	RVOT/Pericardial	13.7	M	Heart transplantation	12 mo, NED
46	2021	Püsküllüoglu M	30/M	RA	82	B	Surgery + CT	26 mo, DOD
47	2021	Matsunaga F	57/F	Interatrial septum	5	_	Surgery	6 mo, AWD
48	2022	Alirezaei T	29/F	LA	4.5	M	Surgery + RT	15 mo, DOD
49	2022	Zhou AL	59/M	LA	5.5	M	Surgery	4 mo, NED
50	2022	Borg B	19/F	RV	5	M	Surgery + CT	12 mo, NED
51	2022	Eqbal AJ	19/M	RV	8.9	_	Surgery + TVP, CT	_
52	2023	Irfan H	25/M	LV	_	_	_	_

RV, right ventricle; RA, right atrium; LV, left ventricle; LA, left atrium; RVOT, right ventricular outflow tract; MV, Mitral valve; M, monophasic; B, biphasic; TVR, tricuspid valve replacement; CT, chemoradiotherapy; RT, radiotherapy; DOD, died of disease; NED, no evidence of disease; AWD, alive with disease.

## Conclusions

The current study reported a rare case of primary cardiac synovial sarcoma presenting in the right ventricle and the tricuspid valve in a pregnant woman. The tumor was complete excised through surgery, and the fetus has survived. The patient was still alive at 30-month follow-up. At present, there are no reported cases of pregnant women with cardiac synovial sarcoma who have underwent cardiac surgery and successfully delivered a surviving baby. Although limited, our contribution provides further data on the management of this rare malignant tumor and cardiac surgery during pregnancy.

## Limitations

We removed the tumor in peacemeal rather than enbloc. Due to the mass almost completely occupies the opening of the tricuspid valve, we removed part of it first before we can proceed to the next section, followed by removal of involved muscle bands and the discrete fragments in the intermuscular space.

## Data Availability

The original contributions presented in the study are included in the article/Supplementary Material, further inquiries can be directed to the corresponding author.
